# Fit for Work and Life—an eight-week program for improvement of functionality and quality of life: A two-stage study

**DOI:** 10.1007/s40211-022-00415-2

**Published:** 2022-04-15

**Authors:** Kunigunde Pausch, Katrin Blanke, Verena Niederberger, Sarah Egli, Michael Rufer, Vladeta Ajdacic-Gross, Sebastian Olbrich, Mario Müller

**Affiliations:** 1 Psychiatric University Hospital Zurich, Militärstr. 8, 8021 Zurich, Switzerland; 2 Triaplus Integrated Psychiatry Uri, Schwyz and Zug, 6317 Oberwil-Zug, Switzerland

**Keywords:** Work integration, Psychiatric patients, Healthcare research, First labor market, Quality of life, Berufliche Integration, Psychiatrische Patienten, Gesundheitsforschung, Erster Arbeitsmarkt, Lebensqualität

## Abstract

**Background:**

The current two-stage study focused on work integration and quality of life of patients in an acute psychiatric day care unit. There is evidence that a longer absence from work due to illness negatively affects job retention, life satisfaction and clinical prognosis. Furthermore, there are individual supportive methods that proved to be effective in work integration. We therefore developed a specific group program Fit for Work and Life (FWL) for patients in an acute psychiatric day care unit focusing on work integration in the first labor market (in contrast to work in institutions for people with disabilities/second labor market).

**Methods:**

Between 2018 and 2020, 62 patients (intervention group; IG) were enrolled in an 8‑week prospective job integration program and compared to 74 patients (control group; CG) who received treatment as usual (partly retrospective survey). Patients of both groups held a job when entering treatment. Main outcome was defined as their working status 4 weeks after the end of treatment as well as self-reported life satisfaction.

**Results:**

At the end of treatment (i.e. the week prior to discharge), the IG participants reported higher overall life satisfaction as well as higher health-, self- and living condition-related satisfaction than controls. Functional and clinical improvement during treatment was linked to subsequently returning to work. Functional improvement was further linked to higher life satisfaction. Mediational analysis revealed an indirect path from functional improvement on life satisfaction via working status, i.e. the higher functional improvement during treatment, the higher the chance of successfully returning to work, which in turn increased life satisfaction.

**Conclusion:**

Our findings suggest that programs such as FWL are useful interventions for employed patients to improve reintegration into work and life and to help to increase life satisfaction.

## Abstract (de)

**Hintergrund:**

Im Fokus der aktuellen 2‑stufigen Studie standen die berufliche Integration und Lebensqualität von Patienten einer akutpsychiatrischen Tagesklinik. Es gibt Evidenz dafür, dass eine krankheitsbedingte längere Abwesenheit von der Arbeit sich negativ auf den Erhalt des Arbeitsplatzes, die Lebenszufriedenheit und die klinische Prognose auswirkt. Außerdem stehen individuelle Unterstützungsmethoden zur Verfügung, die sich als für die berufliche Integration wirksam erwiesen haben. Daher entwickelten die Autoren eine spezielles Gruppenprogramm „Fit for Work and Life“ (FWL), das sich an Patienten in einer akutpsychiatrischen Tagesklinik richtet und den Schwerpunkt auf die berufliche Integration in den ersten Arbeitsmarkt (im Gegensatz zur Beschäftigung in Einrichtungen für Menschen mit Behinderungen/zweiter Arbeitsmarkt) legt.

**Methodik:**

Zwischen 2018 und 2020 wurden 62 Patienten (Interventionsgruppe, IG) in ein 8‑wöchiges prospektives berufliches Integrationsprogramm aufgenommen und mit 74 Patienten verglichen (Kontrollgruppe, KG), die in herkömmlicher Weise behandelt wurden (partiell retrospektive Erhebung). Bei Beginn der Behandlung hatten die Patienten beider Gruppen eine Arbeitsstelle inne. Als Hauptendpunkt wurden der berufliche Status 4 Wochen nach Behandlungsende sowie die Lebenszufriedenheit nach eigenen Angaben definiert.

**Ergebnisse:**

Am Ende der Behandlung (d. h. in der Woche vor der Entlassung) gaben die Teilnehmer der IG eine höhere Gesamtlebenszufriedenheit und eine höhere Zufriedenheit in Bezug auf die Gesundheit, die eigene Person und die Lebensbedingungen an als die Kontrollen. Die funktionelle und klinische Verbesserung während der Behandlung stand im Zusammenhang mit der anschließenden Wiederaufnahme der Arbeitstätigkeit. Darüber hinaus war die funktionelle Verbesserung mit einer höheren Lebenszufriedenheit verknüpft. Die Mediationsanalyse ergab einen indirekten Pfad von der funktionellen Verbesserung zur Lebenszufriedenheit über den beruflichen Status, d. h., je größer die funktionelle Verbesserung während der Behandlung war, umso größer war die Chance auf eine erfolgreiche Wiederaufnahme der Arbeitstätigkeit, was wiederum die Lebenszufriedenheit erhöhte.

**Schlussfolgerung:**

Die vorliegenden Ergebnisse sind ein Hinweis dafür, dass Programme wie FWL nützliche Interventionen für berufstätige Patienten darstellen, um die Reintegration ins Arbeitsleben zu verbessern und so zur Erhöhung der Lebenszufriedenheit beizutragen.

## Introduction

There is evidence that a longer absence from work due to illness negatively affects job retention, life satisfaction and especially clinical prognosis [1–3]. Work mostly has positive effects on mental health. Not having a job can worsen mental health [4]. Mental illnesses can have significant negative effects on the work situation [5]. Issuing lengthy disability certificates can be a dangerous intervention that can lead to permanent illness and eventually permanent disability. The loss of autonomy and independence, waiting in uncertainty, powerlessness in the face of institutions eventually lead to stigmatization and exclusion. This results in a radical change of self-image, leads to perceived worthlessness and significant limitations (financial, social, professional). The aim of the qualitative study by Lännerström et al. [6] was to describe, analyze and understand the experiences of long-term sick people. Participants described that their lifeworld changed when they went on the long sick leave. Individuals who were on long-term sick leave experienced this process as very negative. These negative experiences were among other things related to the consequences of work interruption and the consequences of social security regulations. In many European countries, long-term absences account for a large proportion of the days reimbursed as sick leave. This group is growing and is often made up of women. This leads to isolation and inactivity [7]. After inpatient psychiatric treatment, patients are more likely to lose their jobs and the patients have a worse clinical prognosis than patients who stay in the work process [8]. To avoid such a development, it is important to identify specific factors and thus have greater weight on work integration [9]. The S3 guideline “Psychosocial therapies for severe mental illnesses” as well as previous studies define occupational rehabilitation as psychosocial interventions aimed at improving the work and employment situation of mentally ill people, taking into account the preferences of those affected [10, 11]. Employees in Germany with mental illnesses are on sick leave for around 35 days, which is significantly longer than employees with physical illnesses. This difference has increased considerably in recent years. By 2017, this difference had almost tripled [12]. Apart from individual suffering, the economic burden to society is immense [9, 13, 14]. About one third of the European working population is affected by common mental disorders, which are a leading cause of sick days in Sweden and other countries [15].

Specific programs have been proven to be effective in the area of work integration/supported employment in the form of group therapies/modules for patients with mental impairment [16–20]. This was for example shown by Muschalla et al. [21] with their behavior therapy-oriented work group focusing on fear of work in inpatients. Moreover, patients with a chronic mental illness often have a poorer ability to work. Consequently, it is important that treatment focusses on the ability to work [22]. Linder and colleagues [23] observed overall difficulties in resuming work life after a long absence from working place in a sample of patients with 55% psychiatric–somatic comorbidity. Reker and colleagues [11] pointed out social consequences and long-term effects of mental disorders to be crucial in terms of sick leave, unemployment rates and early retirement. Research on competitive employment showed that supported employment leads to long-term mental well-being and increased life satisfaction [24–29].

The aim of this project was to study the effects of a newly designed short-term cognitive behavior therapy on work-anxiety and sick-leave in patients with work-related difficulties to close this gap. Therefore, we evaluated this specific 8 week program Fit for Work and Life (FWL) that specifically addressed work reintegration in existing employment conditions or self-employment in the first labor market.

## Methods

### Setting

The FWL is a combination of individual and group therapy as part of the multimodal treatment approach of the Acute Day Clinic (German: Akut-Tagesklinik; ATK) of the Psychiatric University Clinic Zurich. Patients with mental health problems are treated in the ATK on a day-care basis, as an alternative to inpatient treatment. ATK was established in 2010 as an inpatient alternative that provides high-frequency treatment several times a week in group as well as in individual settings. The ATK is open 7 days a week, 365 days a year. Psychiatric patients aged 18–64 years with all diagnoses (except patients with addiction as the main diagnosis) are treated. The ATK offers flexible treatment options such as a reduced therapy program towards the end of the treatment with, for example, simultaneous return to work. On average, the ATK treats over 200 patients per year. The ATK is managed by medical professionals. The idea for the FWL group is based on the senior psychiatrist of the ATK in 2018 on the background of the ATK representing a treatment alternative in lieu of a ward, some patients are still employed or still have a job at the time of admission. In the years from 2014 to 2018 (change of senior psychiatrist in 2014), it was observed that the patients often displayed avoidance behavior regarding the topic of their work. The fact that this avoidance behavior was not effective and did not lead to psychological stabilization was also shown by the fact that these patients (without an appropriate program) did not return to work and became psychologically worse. The FWL program was developed to address this problem. The senior psychiatrist of the ATK and a psychologist, who mainly worked for the supported employment and who was significantly involved in the performance of the FWL program, were responsible for the content of the program.

### Study design: Fit-for-Work-and-Life program

The FWL program is specifically addressed work-related issues in a combination of individual and group settings. The group contents were developed on a behavioral therapy basis and after appropriate literature research by the senior psychiatrist and the psychologist from supported employment. The medical supervised management of the FWL program by the psychologist who mainly worked in supported employment is ideal in combination with the flexible treatment options in the ATK (gradual start of work already during therapy, living in the familiar environment). Weekly group sessions of 90 min with maximal 10 participants were provided with additional individual meetings for 30 min if required.

Contents of the 8 group sessions were as follows:

1. Examination of the individual working biography: elicit individual strengths and skills.
2. Clarify questions concerning social insurance.
3. Identifying individual stressors and find a way to cope with them—1: Identifying stressors, reflecting perceived control, mindful based exercises [30].
4. Identifying individual stressors and find a way to cope with them—2: Strategies and exercises e.g. concerning the meaning of the appraisal of a situation, or how to cope with increased demands of her- and himself or others [30].
5. Communication—1: Recognizing the dynamics of conflicts, reflecting the individual communication, exercises in nonviolent communication [31].
6. Communication—2: How to express needs in the working context, learning to cope with critics, exercises in nonviolent communication [31].
7. Recurrent challenges and difficulties in the workplace—1: Identifying behavior patterns which are necessary to adapt while resuming work, supplementary exercises to prevent burnout [32–34].
8. Recurrent challenges and difficulties in the workplace—2: Additional exercises corresponding to participants needs, e.g. self-perception, gaining a different perspective, lacking appreciation of the manager, coping with pressure [32–34].

In the group setting, the participants’ work biographies can be discussed initially, then social–psychiatric issues are addressed with the support of a social worker. This is followed by communication training with role playing and subsequent situation analysis in relation to difficult work situations. In the individual setting (between psychologist and patient), which each group participant was also offered weekly, individual questions could be dealt with (e.g. biography) and important telephone calls (e.g. contacting employers) could be prepared and, if necessary, also carried out. The group size was generally 10 patients. The last month of recruiting the intervention group (April 2020) fell within the COVID-19 (coronavirus disease 2019) pandemic. The program continued under protective measures with a slightly reduced number of patients (8 patients). Participation in the individual setting was voluntary, as was participation in the group setting. At least three participations in the group program were required for inclusion; otherwise, the program was classified as “not attended”.

The study was approved by the Ethics Committee (EC) of the University of Zurich.

### Study sample

In total 136 patients were included in the two-stage study, whereof 62 gave consent to participate in the program as intervention group (IG) and 74 as control group (CG). The CG was recruited from patients who attended ATK from April 2018 to April 2019 prior to program start. Two thirds of the patients in the CG were recruited at the time of the regular discharge of the ATK (from November 2018). A smaller proportion of patients completed the questionnaires retrospectively—a total of one third of the patients (10 patients in absolute terms). The longest time frame was 6 months because the survey of the control group started retrospectively in November 2018. All “retrospective” patients were contacted by telephone in November 2018 to provide detailed information about the study and to answer the questionnaires. Many patients who were written to in November 2018 to take part in the study as a CG regarding their stay in the ATK from May 2018 did not answer or did not return the form.

It was a two-stage study because the IG consisted of patients who were treated at the ATK from May 2019 onwards (prospective study design). The reason was that the program FWL was not integrated into the ATK program until 2019. The inclusion criteria for both groups (IG/CG) were holding an ongoing job at the time of entry into the ATK or self-employment of at least 20% workload in the first labor market. Patients aged 18–64 years were integrated. Participants of the IG (*n* = 62) were informed about the study before attending the FWL program, all patients who met the inclusion criteria for the study were strongly advised to have at least one consultation with the psychologist conducting the group before beginning the program and those who actually participated received subsequently the same questionnaires and informed consent form as the CG.

### Hypotheses

It was hypothesized that (a) those patients who completed the FWL program would more likely return to work within 4 weeks after discharge from treatment than patients who did not participate in the program. It was further hypothesized that (b) FWL participants report a higher life satisfaction than controls at time of the follow-up. In addition, the association life satisfaction should be researched independently, i.e., it was expected (c) that returning to work successfully on their own was related to higher life satisfaction than being still on sick leave 4 weeks after the program. Then, it was (d) expected that work reintegration was caused by a higher functional level after the therapeutic intervention and therefore serves as intermitting or mediating factor within the pathway between therapeutic outcome and life satisfaction.

### Measures

A two-part questionnaire was conducted, which included the purpose and a description of the study as well as an informed consent form. The first part of the questionnaire asked among other questions whether the participant returned to work for a workload of at least 20% within 4 weeks after leaving their treatment (working status). The second part assessed life satisfaction based on the WHO 8‑item Quality of Life questionnaire (German version), at the time of leaving the ATK (EUROHIS-QoL8 [35]), with questions regarding satisfaction with daily life, health, social network, locus of control etc., which were assessed at discharge from the ATK. Items of the EUROHIS-QoL were inverse coded for sake of a better interpretability, i.e. higher scores reflecting higher satisfaction or quality of life, respectively. The EUROHIS-QoL8 has a good internal consistency (Cronbach’s α = 0.83).

Sociodemographic data, such as age, primary diagnosis, level of functioning and clinical severity, were also taken into account. Sociodemographic, clinical and diagnostic information were all collected using the basic documentation of the psychiatric statistics of the Canton of Zurich “PSYREC” [36], which is an obligatory assessment at the beginning and the end of each treatment at our hospital.

Functioning was measured using the Global Assessment of Functioning (GAF), a 100-point single item observer rated scale with a continuum from mostly impaired (0) to maximal mental health (100). The GAF reflects overall functioning for the past 7 days and was assessed at the beginning (baseline) and at the end of treatment, while the difference in score was used as a measure for functional improvement [37]. The CGI [38] was used as a measure for clinical severity (CGI-S) and improvement (CGI-I). The CGI scales are widely used tools for measuring treatment outcomes in general psychiatry [39] and were originally introduced in psychopharmacological trials [38]. They provide a brief, universal stand-alone assessment of illness severity and its change over time. The severity (CGI-S) and the improvement scale (CGI-I) have a 7-point Likert scale response format. Severity ranges from 1 “not ill at all” to 7 the “extremely ill”, while improvement ranges from 1 “highly possible improvement” to 7 “most severe deterioration”. For the current analyses we inverse-coded the improvement scale for a better interpretability (i.e. higher scores = higher improvement). Ratings of the CGI‑S refer to the past week, ratings of the CGI‑I to the time since the first CGI‑S assessment, i.e., the duration of treatment [40].

### Statistical analyses

Descriptive statistics are provided for sociodemographic and clinical characteristics and for reports on life satisfaction. Frequencies and percentages are reported for categorical variables and means (M) and standard deviations (SD) for continuous variables. To examine differences between groups, we calculated Χ^2^ statistics for categorical variables and one-way analysis of variance (ANOVAs) for continuous variables. To assess whether the study sample is a representative part of the total treatment population, we first compared the study sample with the patients in the treatment-as-usual group on demographic and clinical conditions.

Primary outcome variables were whether the patients returned to a job in the first labor market immediately within 4 weeks after leaving the ATK as well as self-reported life satisfaction at the time of leaving the ATK. Therefore, we examined whether the subgroups of the study sample differed regarding self-reported life satisfaction and work status.

**Fig. 1:**
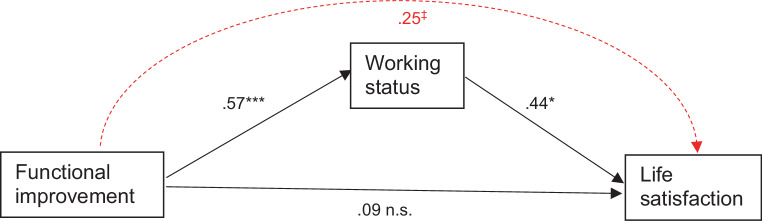
Path modeling the mediation of functioning on life satisfaction via working status. ***p ≤ 0.001; *p ≤ 0.05; ^‡^*p* ≤ 0.10; *n.* *s.* not significant

Working status was examined regarding both direct effects and its role as a mediator between functional improvement and life satisfaction. Therefore, a path model was specified linking functional improvement to life satisfaction through working status (Fig. 1). For this analysis we used the INDIRECT command in MPlus to estimate the indirect or mediated effects. Then, as recommended by Mackinnon et al. [41], the model was re-run with 1000 bootstrap samples to test for the significance of the indirect effect. Bootstrap indirect path coefficient with 95% bias-corrected confidence intervals was calculated. A confidence interval that did not include zero indicated a significant indirect effect [41]. Path modeling was conducted using MPlus v7.11 [42].

All statistical analyses were conducted using STATA/SE 16 [43].

## Results

The final study sample did not differ from the remaining, i.e. not participating, patients’ population of the acute day hospital in neither sociodemographic features, such as sex, age or education, nor their primary psychiatric diagnosis at the end of treatment (not tabulated). However, the study sample had slightly higher clinical (mean difference = 0.4, *p* = 0.02) and functional improvements (mean difference = 5.2, *p* = 0.028) during treatment than others.

In total, 38 patients (of the 62 patients in the whole IG) visited the FWL group three times or more and 31 patients also had a completed questionnaire at the end. Of the total control group (*n* = 74), a total of 30 completed questionnaires were returned. More than half (*n* = 20) of the IG received an additional psychological one-to-one individual job coaching session, which was offered and recommended to each FWL group participant at the beginning of the group.

All conditions were met by 16 IG participants, i.e. at least three participations, individual coaching as well as the completed questionnaire, while 15 met these conditions as well except for the coaching session. Further differentiation of participation (e.g., participants who participated more than five times) was not made due to the small collective. Of the 31 patients, 19 participated in the program between six and ten times. Four patients each participated in the program three, four and five times (i.e. a total of 12 patients between three and five times). On average, the participants took part in the group five to six times.

**Fig. 2:**
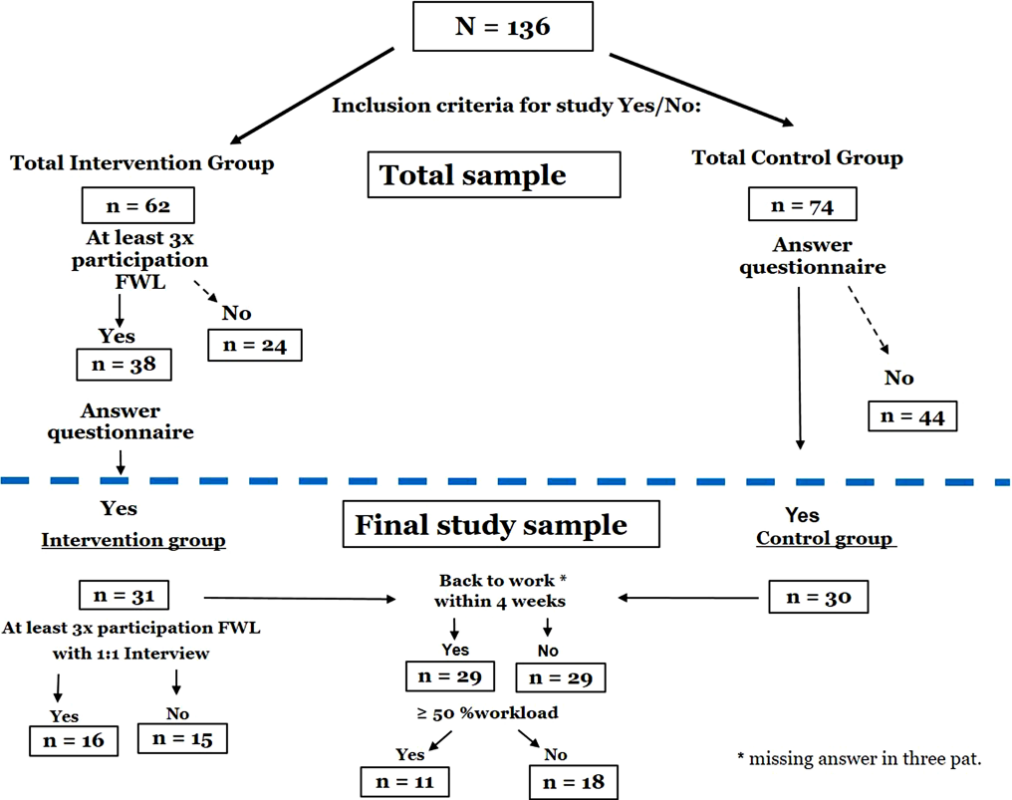
Sampling flow chart: group division

Overall, acceptance among participating patients was high, it has to be noted that of the 62 patients meeting the inclusion criteria for the intervention group, only 38 patients attended the group at least three times and 31 patients completed the questionnaires (Fig. 2). The reasons why only about 20 of the 31 patients requested an individual setting were that some of the patients received notice of job termination during the program and therefore had little motivation to participate in the program, and further that the patients were already able to clarify issues that were important to them in the group setting.

The professions of the patients were heterogeneous. They ranged from actors to vocational school teachers to butchers and lawyers. Sick leave before admission ranged from a few days to 2 years (intervention group) or 3 years (control group). On average, patients were on sick leave between 4 and 10 weeks before admission.

No differences in sociodemographic features were found between IG and CG. CGs, however, had better baseline functioning (at the beginning of the treatment) than the IG, while the latter, in contrast, showed better clinical improvement during treatment (Table 1). At intervention subgroup level (i.e., additional interview vs. not), no significant group differences in neither sociodemographic nor clinical variables were found.

**Table 1:** Sociodemographic and clinical features of the CG versus IG as well as intervention subgroups

		Control group *n* = 30	Intervention group (plus survey)			Group comparisons	
			Total *n* = 31	1:1-interview *n* = 16	No interview *n* = 15	IG vs. CG	Subgroups^a^
Sex female; %	–	50.0	51.6	50.0	53.3	0.900	0.975
Age; M ± SD	–	39.9 ± 12.2	34.7 ± 11.0	33.7 ± 10.3	35.8 ± 12.0	0.085	0.203
Education; col%	Low	6.7	10.0	–	20.0	0.891	0.325
	Medium	56.7	53.3	53.3	53.3		
	High	36.7	36.7	46.7	26.7		
Diagnosis; col%	Psychotic disorder	3.3	9.7	6.3	13.3	0.698	0.835
	Affective disorders	70.0	71.0	68.8	73.3		
	Anxiety disorders	20.0	16.1	18.8	13.3		
	Personality disorders	6.7	3.2	6.3	–		
	Other disorders	–	–	–	–		
Overall functioning; M ± SD	–	54.1 ± 11.7	49.3 ± 5.9	49.7 ± 6.6	48.8 ± 5.1	0.046*	0.134
Functional improvement; M ± SD	–	14.4 ± 13.2	20.4 ± 12.3	22.4 ± 12.8	18.2 ± 11.8	0.074	0.137
Clinical severity at baseline; M ± SD	–	5.7 ± 0.6	5.9 ± 0.7	5.9 ± 0.8	6.0 ± 0.7	0.254	0.463
Clinical improvement (reverse coded); M ± SD	–	3.4 ± 1.0	2.8 ± 0.7	2.9 ± 0.6	2.7 ± 0.9	0.018*	0.051

*IG* intervention group, *CG* control group, *M* mean, *SD* standard deviation, *col%* column percent

*p ≤ 0.05

^a^Subgroups = CG + IG_interview_ + IG_no interview_

The IG reported higher overall life satisfaction than the CG (Table 2). Only three items were found to be distinctive, i.e., in favor of a higher satisfaction in the IG: health-related and self-related satisfaction as well as satisfaction with conditions of living place. Those with an intervention plus additional face-to-face interview were more satisfied with conditions of living place and reported higher quality of life than controls did.

**Table 2:** Satisfaction and quality of life of the CG versus IG as well as intervention subgroups

	Control group *n* = 30	Intervention group (plus survey)			Group comparisons	
		Total *n* = 31	1:1 interview *n* = 16	No interview *n* = 15	IG vs. CG	Subgroups^a^
Do you have enough energy for everyday life? (M ± SD)	2.8 ± 0.9	3.1 ± 0.9	3.2 ± 0.9	3.1 ± 1.0	0.223	0.445
Have you enough money to meet your needs? (M ± SD)	3.1 ± 1.4	3.1 ± 1.1	2.9 ± 1.0	2.9 ± 1.3	0.919	0.844
How satisfied are you with your health? (M ± SD)	2.4 ± 0.9	2.9 ± 0.9	2.9 ± 1.0	2.9 ± 0.9	0.021*	0.069
How satisfied are you with yourself? (M ± SD)	2.4 ± 0.9	3.0 ± 1.0	3.0 ± 1.2	3.1 ± 0.9	0.025*	0.082
How satisfied are you with your ability to perform your daily living activities? (M ± SD)	2.8 ± 1.0	2.9 ± 1.1	2.9 ± 1.2	3.0 ± 0.9	0.520	0.769
How satisfied are you with your personal relationships? (M ± SD)	3.2 ± 1.1	3.4 ± 0.9	3.6 ± 1.1	3.2 ± 0.7	0.395	0.424
How satisfied are you with the conditions of your living place? (M ± SD)	3.3 ± 1.2	4.2 ± 0.9	4.4 ± 0.9	3.9 ± 0.8	0.003**	0.006** CG < IG_int_**
How would you rate your quality of life? (M ± SD)	2.9 ± 0.8	3.3 ± 1.0	3.7 ± 0.8	2.9 ± 1.1	0.100	0.015* CG < IG_int_*
Total sum score (= overall life satisfaction) (M ± SD)	22.7 ± 5.4	25.3 ± 4.9	25.6 ± 4.9	25.0 ± 5.0	< 0.050*	0.140

*IG* intervention group, *CG* control group, *M* mean, *SD* standard deviation

*p ≤ 0.05, **p ≤ 0.01

^a^Subgroups = CG + IG_interview_ + IG_no interview_

None of the intervention subgroups differed from controls or between each other in their working status (not tabulated). There was no significant difference between the CG and IG in terms of return to work at the first job 4 weeks after leaving ATK. Overall, working status was neither associated to sociodemographic features nor to psychiatric diagnoses (not tabulated). However, being back to work was associated with better overall functional (*p* < 0.001) and clinical improvement (*p* = 0.018) (not tabulated). At time of admission, 26 persons in the IG and 22 in the CG had employment of at least 80%. Solely functional improvement was higher in both working subgroups^b^ compared to those who did not work but not between them.

Those who were back to work reported higher overall life satisfaction, specifically in areas such as health, themselves/self-concept, ability to perform daily life activities, conditions of living place as well as quality of life (Table 3). No specific subgroup differences between full- and halftime workload were found.

**Table 3:** Satisfaction and quality of life in relation to working status (missing answer in three patients)

	Not back to work *n* = 29	Back to work			Group comparisons	
		Total *n* = 29	Work load less than 50% *n* = 18	Workload 50% or more *n* = 11	Back to work within a month vs. not	Working subgroups^a^
Do you have enough energy for everyday life? (M ± SD)	2.7 ± 1.0	3.2 ± 1.0	3.3 ± 1.0	2.9 ± 1.0	0.077	0.159
Have you enough money to meet your needs? (M ± SD)	2.9 ± 1.1	3.3 ± 1.4	3.3 ± 1.4	3.3 ± 1.5	0.150	0.401
How satisfied are you with your health? (M ± SD)	2.4 ± 0.9	2.9 ± 0.9	2.9 ± 0.9	2.9 ± 0.8	0.014*	0.072
How satisfied are you with yourself? (M ± SD)	2.4 ± 0.9	2.9 ± 1.0	2.8 ± 0.9	3.1 ± 1.1	0.036*	0.095
How satisfied are you with your ability to perform your daily living activities? (M ± SD)	2.5 ± 0.9	3.1 ± 1.0	3.2 ± 0.9	3.1 ± 1.1	0.006**	0.024* NW < W_low_*
How satisfied are you with your personal relationships? (M ± SD)	3.2 ± 1.1	3.4 ± 1.1	3.2 ± 0.9	3.6 ± 1.2	0.367	0.470
How satisfied are you with the conditions of your living place? (M ± SD)	3.3 ± 1.2	4.3 ± 0.7	4.3 ± 0.5	4.3 ± 0.9	< 0.001***	0.001** NW < W_low_* NW < W_high_*
How would you rate your quality of life? (M ± SD)	2.8 ± 0.9	3.3 ± 0.9	3.2 ± 0.9	3.4 ± 1.0	0.027*	0.095
Total sum score (= overall life satisfaction) (M ± SD)	21.8 ± 4.8	26.1 ± 5.2	26.2 ± 5.5	25.9 ± 5.3	0.001**	0.006** NW < W_low_** NW < W_high_*

*IG* intervention group, *CG* control group, *NW* not back to work, *W*_*low*_ back to work with work load less than 50%, *W*_*high*_ back to work with work load 50% or more, *M* mean, *SD* standard deviation

*p ≤ 0.05, **p ≤ 0.01, ***p ≤ 0.001

^a^subgroups: Work load less than 50%/workload 50% or more

Moreover, from those life satisfaction (LS) items that were linked to work (Table 3), three, i.e., health (*p* = 0.018), self-concept (*p* = 0.016) and quality of life (*p* < 0.001), as well as the total LS score (*p* = 0.005) were linked to functional improvement as well (not tabulated). Therefore, in order to assess whether those single associations might be part of a more complex interplay, whereby functioning affects life satisfaction indirectly (via working status) rather than directly, a path model was specified to test for mediation effects. For the sake of simplicity, the LS total score was chosen as an outcome variable.

Fig. 1 displays the results of the mediation analysis. The results revealed the functioning–life satisfaction association to be fully mediated by the effect of working. This was indicated not only by a decrease in the strength of the direct association (r_difference_ = 0.25; which alone would indicate a partial mediation) but also by the loss of significance (which in addition indicates full mediation). The indirect effect estimate was quantified as β = 0.25 at trend level significance (*p* ≤ 0.1).

## Discussion

The current study aimed to show that the special group program on work integration with individual job-coaching as specifically designed and established in the ATK of the PUK Zurich might lead to higher LS, especially to higher LS concerning the place of living in patients with a job at the time of entry compared to patients who did not participate in the program. The setting was generally well received and accepted by the participants in the IG (*n* = 31) and it as considered feasible by the patients although it should be mentioned that only 38 patients of the whole IG (*n* = 62) participated in the group program at least three times and 31 patients filled in the questionnaires. The most common reason for nonparticipation was that the patients still felt overwhelmed with the topic of work and did not want to participate. There were some more program-independent reasons for early dropout from the group or for a later start in the group. These reasons include: First, most often many of the cases with low participation rates can be justified by the fact that their job was terminated by the employer or they themselves quit their job. Thus, for these patients, participation in the group was no longer purposeful—or in the case of a termination, then too burdensome. The second very frequent reason was early departure from the ATK (e.g., early transfer to the continuing day clinic at the same hospital). Furthermore, the group was often fully booked (limited number of participants: 8–10 patients), which is the reason why individual patients were only able to participate later. With the start of the group in May 2019, there were some cases who had already completed half of the ATK program and were therefore only able to participate a few more times.

Overall, participants expressed satisfaction with the FWL program to the treatment team. Group sessions were accepted as well as individual sessions. Feasibility was described as good by the lead psychologist. However, the experience showed that the program is only suitable for or patients who still have employment and not for patients who have already received a job termination during the first weeks of the ATK (which was probably also due to the fact that patients were often already on sick leave for several weeks before entry).

Individual setting was offered and recommended to all patients, but it was not taken up from all of the patients. From the authors’ point of view, it still makes sense to include patients, even if they only took part three times and even if they had no individual special setting because the plan for resuming work was discussed with all new FWL participants at the first appointment. After three sessions, they should at least have a rough idea of how to proceed in terms of contacting the employer and the necessary clarifications before returning to work. All in all, the team was very committed to motivate the patients with a job for regular participation in the FWL program. The acceptance of the patients who participated was very high. It remains to be said that job integration is a sensitive topic for which some patients were not yet ready at the beginning of the ATK. Nevertheless, the authors consider such a program for motivated patients to be very useful. All eligible patients should also be informed about such a program, at least in an individual setting.

There was no significant difference between the CG and the IG in terms of return to work at the first job 4 weeks after leaving ATK.

The reason for the lack of difference in terms of return to work between the control and intervention groups could be, on the one hand, the relatively small number of patients and, on the other hand, the relatively short observation period of 4 weeks after discharge. Perhaps the return to work among program participants 3 months after leaving would have been significantly higher than in the CG. Some of the patients transferred to another treatment (day clinic/inpatient). Returning back to work was, however, found to act as a trigger within the association between functional improvement during treatment and life satisfaction, i.e., successfully returning to work as a consequence of increased functioning leads to higher life satisfaction. The acute day clinic provided the appropriate setting for this intensive job integration training, as the patients were able to remain in their home environment despite intensive therapy, thus, lowering the threshold for transition and return to everyday life and working life more than would have been the case with inpatient treatment. In addition to the ATK, supported employment is also available at the Centre for Social Psychiatry of the PUK Zurich (Location Militärstraße). There was already intense cooperation between the two departments before the group was established.

Although we could not show that the program had a positive impact on work integration compared to the control group, patients who participated in the program seemed to benefit from the intervention in terms of a higher quality of or satisfaction with life, respectively. Since patients with improved functioning more likely returned to work and showed higher life satisfaction, it was found that functioning level indirectly influenced quality of life, mediated by the factor work. Why satisfaction with the place of living was the highest remains unanswered. The literature is of little help. It is conceivable that the city of Zurich itself, as a city with one of the highest qualities of life in the world, plays a central role in this.

An earlier study conducted by Viering et al. [27] already dealt with the satisfaction of patients with individual placement and support (IPS). The overall satisfaction of the participants with the IPS services was very high. Furthermore, client satisfaction and symptom severity were inversely associated. Studies have shown that somatic and psychological comorbidities, education and socioeconomic often fundamentally influence the ability to work [44]. The number of patients with difficulties in returning to work after prolonged illness has increased in most European countries, which has been shown by several studies [23, 45]. Supported employment concepts such as the Individual Placement and Support (IPS) model were developed to reintegrate unemployed people with mental disorders back into work (Kawohl et al. [46]). The largest European multicenter randomized controlled trial to date on the effectiveness of supported employment (EQOLISE—effectiveness of supported employment) was carried out in six European centers, including the Psychiatric University Hospital Zurich until 2005. It could be shown that the intervention “individual placement and support” was superior to traditional integration programs. The study by Jäger et al. [47] showed that the sustainability of supported employment for people with severe mental disorders in terms of maintaining employment and income is limited if job coaching is not continued. The FWL program also contains many psychotherapeutic elements combined with strategies from supported employment. This could be achieved through the staffing of the program (supervised by a medical doctor and led by a psychologist who works mainly in supported employment). The lack of work reintegration of patients has significant effects on their own well-being and recovery. It is also worth mentioning the considerable costs incurred by the prolonged illness (direct [treatment] costs) as well as by the lack of work for society (indirect costs). Disability pensions are the consequence. Occupational dysfunction associated with psychiatric disorders can also lead to social isolation and poverty [48]. Mental illnesses are the most common cause of disability pensions in Switzerland today. It is true that the number of new “IV” (German: *Invalidenrente* = disability pension) pensions awarded each year has been halved since 2003 and the proportion of IV pensioners in the insured population has fallen continuously over the last 10 years. However, if we look at individual causes of disability and individual age groups, a different picture emerges: the number of new pensions for young people is stagnating and the proportion of IV retirements due to mental illness is increasing among 18–29 year olds [49]. A number of previous studies have examined various predictors of the award of a disability pension. The aim of Wallmann et al. [50] was to test the effectiveness of sick leave balance as a predictor of the award of a disability pension in a dataset based on individuals from the general population who had been observed long-term. Sick leave balance was the most important predictor of the probability of receiving a disability pension, even after accounting for the influences of other variables affecting the outcome [50]. The desire to work is something human and is also present in many people with mental illness. However, every day that someone does not work makes it more difficult for them to return to the job market. Many employers are very concerned about employees who are mentally ill. In this age, it is crucial to understand how work integration takes place after or during a mental illness. According to Holinger and Schoppmann [51], gainful employment is more than just a necessary means of subsistence. It is important for identity, for finding meaning and for self-expression, and not least for social status. Personal recovery means leading a meaningful and self-determined life, regardless of whether symptoms persist or not. Work should be an integral part of personal recovery. Focusing on this is a task for all mental health professionals and not just a task for disability insurance. There is limited scientific evidence of comparable programs in similar treatment settings [28]: The design of the FWL program in four parts is conducive to flexible work integration during psychiatric treatment.

### Limitations

The study has a number of limitations that have to be mentioned. First, we have used a convenience sample for studying the effects of a working integration program since the program FWL was developed only recently. All patients with a job in the primary labor market were included. The IG was recruited prospectively since May 2019. At the time of recruiting (prospective survey for IG) the CG (April 2018), the program was not yet established (partly retrospective survey for CG). Therefore, it was a two-stage study. Second, given the relatively long observation period of one year for the CG and about one year for the patient group, the total sample of *n* = 136 patients was rather small. It might be possible that a higher number of cases would result in the expected effect of a significantly higher return to work following treatment for the IG. Third, the observation period for the return to work of only 4 weeks after discharge is relatively short. It is generally known that returning to work after psychiatric treatment is difficult and many patients lose their jobs [9]. This is especially the case for inpatients, also because psychosocial support for this group of patients at the clinic/outpatient clinic interface is often inadequate. In recent years, awareness of the interaction between work and mental health has increased greatly. However, the group of patients suffering from mental illnesses requiring hospitalization and their possible return to work has been neglected [52]. Fourth, the minimum participation (when the program is considered attended) of only three times is low. This could also be a reason why there was no difference between the CG and IG in terms of return to work 4 weeks after exit. Even though the authors concluded that significant success in terms of return to work could be achieved after only three appointments, it can be assumed that a higher level of participation is more likely to lead to a return to work. However, the number of patients in the current study is too small to be able to prove this effect significantly. For future studies, it might be useful to expand the inclusion criteria in order to integrate patients with an interest in work-specific questions as well as to measure life satisfaction and the general level of functioning.

## Conclusion

The 8‑week program FWL consisting of group and individual treatment seems to be a useful program for patients who are still working during time of treatment. Participation improves life satisfaction in general and especially with regard to the “place of living”, life satisfaction “with oneself” and “satisfaction with one’s own health”. Patients with improved clinical functioning levels returned to work more often, which in turn leads to higher life satisfaction. The program, which has currently (in 2022) been running for 4 years, is now an integral part of the ATK. It is very much appreciated by patients.

Further and larger studies on work integration and/or quality of life in the field of social psychiatric care should be conducted, even though the study setting is very complex to implement. Thus, further programs led by appropriate professionals are important, should be established and should also be scientifically investigated in terms of their content.
